# Light-Responsive Actuators Based on Graphene

**DOI:** 10.3389/fchem.2019.00506

**Published:** 2019-07-17

**Authors:** Yuan-Yuan Gao, Bing Han, Wen-Ya Zhao, Zhuo-Chen Ma, Yong-Sen Yu, Hong-Bo Sun

**Affiliations:** ^1^State Key Laboratory of Integrated Optoelectronics, College of Electronic Science and Engineering, Jilin University, Changchun, China; ^2^State Key Laboratory of Precision Measurement Technology and Instruments, Department of Precision Instrument, Tsinghua University, Beijing, China

**Keywords:** light-responsive, actuators, graphene, graphene oxide, photothermal effect

## Abstract

As a typical 2D carbon material, graphene, that possesses outstanding physical/chemical properties, has revealed great potential for developing soft actuators. Especially, the unique properties of graphene, including the excellent light absorption property, softness, and thermal conductivity, play very important roles in the development of light-responsive graphene actuators. At present, various light-driven actuators have been successfully developed based on graphene and its derivatives. In this mini review, we reviewed the recent advances in this field. The unique properties of graphene or graphene-related materials that are of benefit to the development of light-driven actuators have been summarized. Typical smart actuators based on different photothermal/photochemical effects, including photothermal expansion, photothermal desorption, photoisomerization, and photo-triggered shape memory effect, have been introduced. Besides, current challenges, and future perspective have been discussed. The rapid progress of light-responsive actuators based on graphene has greatly stimulated the development of graphene-based soft robotics.

## Introduction

Graphene is a single-atom-thick 2D material with carbon atoms arranged in honeycomb crystal lattice. The unique structure enables graphene to have high electron mobility, high thermal conductivity, large specific area, high transparency, and good flexibility (Medhekar et al., 2010). However, the weak chemical activity and the problems in mass production of pristine graphene greatly impede its practical application. So that, various alternative strategies are investigated to tailor the physical and chemical properties (Cheng et al., 2017). Graphene oxide (GO) that possesses abundant oxygen-containing groups (OCG, e.g., carbonyl, carboxyl, and hydroxyl) onto graphene lattice is a non-conductive but hydrophilic material. GO can be well-dispersed in many aqueous solutions and shows strong interaction with various guest molecules, revealing great potential for cutting-edge applications, for instance, sensors and actuators.

As an essential component of the intelligent system, actuators can be categorized according to diverse stimuli (Ariga et al., 2016), such as light (Han et al., 2016; Han B. et al., 2018), moisture (Han et al., 2015; Cheng et al., 2016), electric (Zhu et al., 2019), solvent (Zhang et al., 2019), pneumatic (Wang et al., 2018), and so on. Among these methods, light-driven strategy is more appealing for the remote and untethered control. Optical energy contains different information, including light intensity, frequency, polarization, and spot size, leading to the flexible manipulation of actuators. However, most light-driven actuators have relatively low energy conversion efficiency, especially compared to electron-responsive ones. The addition of graphene-related materials can overcome this problem, because of their efficient light absorption and outstanding heat conductivity (Nair et al., 2008; Prasher, 2010). As a result, graphene has emerged as a promising host or additives for light-driven actuators.

In this paper, we summarized the recent development of graphene-based light-driven actuators. The advantages of graphene related materials in preparing optical sensitive devices are highlighted. Typical light-to-work conversion mechanism, including photothermal expansion, desorption, photoisomerization, and photo-induced shape memory effect, are reviewed. In addition, the applicability and limitations of this field have been discussed briefly.

## Unique Properties of Graphene for Light-Driven Actuators

Graphene has been proven to be a promising host material for light-driven actuators. First, graphene has a broadband absorption of light, and a graphene single layer can absorb ~2.3% of white light (Nair et al., 2008). External light energy forces the vibration of phonon and the material exhibits a rapid temperature increment. High transparent photothermal devices can be realized due to the ultra-thin structure (Wu et al., 2011), excellent light absorption property and high photothermal conversion efficiency of graphene. Second, actuators produce mechanical deformation reversibly, so good flexibility and high robustness are strictly demanded. Graphene has a superior flexibility, which can be designed into different structures, such as fibers (Meng et al., 2015), films (Liu et al., 2016), and foams (Hu et al., 2016). The Young's modulus of graphene can reach 1 TPa, and the intrinsic strength is as high as 130 GPa (Novoselov et al., 2012). Besides, graphene is reported to have a negative coefficient of thermal expansion (CTE, −6 ppm·K^−1^, 300 K) (Yoon et al., 2011), so when it is combined with other materials that have a positive CTE, the bilayer will bend toward the graphene side due to the photothermal effect. It is worth mentioning that GO demonstrates a more apparent negative CTE, deriving from the adsorption/desorption of water molecules associated with temperature changes (Zhu et al., 2012). Numerous actuators have been made based on the photothermal desorption effect (Mu et al., 2015; Chen L. et al., 2017; Han D. D. et al., 2018). The excellent electronic conductivity and thermal conductivity (up to 5,000 W·m^−1^·K^−1^) (Balandin et al., 2008) can further facilitate the actuation process, exhibiting fast and large bending performance. The raw material of graphene, that is graphite, is quite abundant on earth. Graphene also has a good stability under ambient condition. Compared with other photothermal materials, such as metal nanoparticles (NPs) and dyes, graphene material is much more cost-effective and stable. Taking advantages of these exceptional properties, graphene, and its derivatives are promising for developing light-driven actuators.

## Light-Responsive Actuators Based on Graphene and Its Derivatives

**Figure 1:**
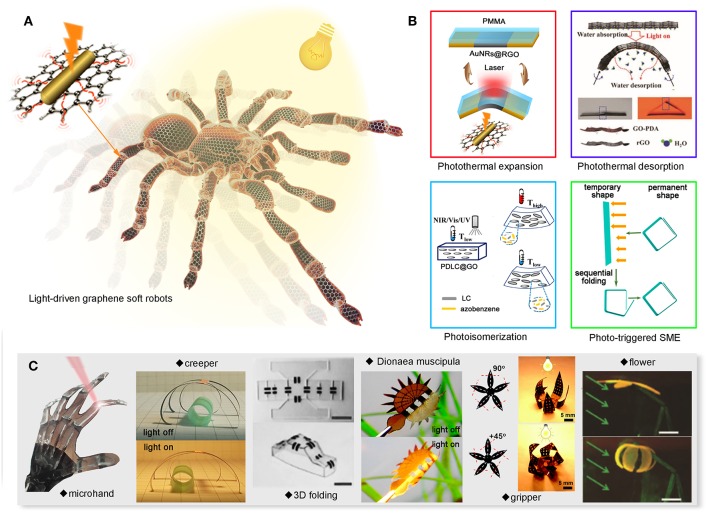
Graphene-based light driven actuators. **(A)** General concept of light-driven graphene soft robot. **(B)** Four typical mechanisms of light-driven actuators. **(C)** Examples of light-driven graphene actuators. Reproduced from Han et al. (2019) with permission of WILEY-VCH. Reproduced from Mu et al. (2015) with permission of American Association for the Advancement of Science. Reproduced from Cheng et al. (2015) with permission of American Chemical Society. Reproduced from Chen T.T. et al. (2017) with permission of American Chemical Society. Reproduced from Han D. D. et al. (2018) with permission of WILEY-VCH. Reproduced from Tang et al. (2017) with permission of WILEY-VCH. Reproduced from Deng et al. (2018) with permission of the Royal Society of Chemistry. Reproduced from Ma et al. (2018) with permission of WILEY-VCH.

Light-responsive actuators can effectively convert light into mechanical work. It has been extensively studied and applied in autonomous systems, robotics and biomedical science. Among these works, the mechanism of energy conversion can be classified into several modes. In this section, we briefly summarized the typical energy conversion strategies of graphene-based light-driven actuators (Figure 1).

### Photothermal Expansion

As we mentioned above, graphene possesses a negative CTE. In combination with a material of large and positive CTE, the asymmetric expansion of bilayer structures might occur during temperature rise. The different volume change of the two materials leads to a directional bending. The light energy converts to the mechanical deformation through the photothermal expansion strategy. Han et al. produced a graphene actuator by integrating a polymethyl methacrylate (PMMA) layer with graphene and gold nanorods composites (Han et al., 2019). The presence of gold nanorods endows the final actuator with wavelength-selectivity. Spider robot made of this structure can walk freely by alternately irradiating laser spots on their legs. Deng et al. made an actuator based on a sandwiched structure of poly(vinylidenefluoride)/laser induced graphene/polyimide (PVDF/LIG/PI) (Deng et al., 2018). In this work, PVDF has a large CTE; however, LIG and PI are inert to temperature change. LIG can form highly aligned structures, so that the bending direction is predictable and the actuator can achieve programmable complex shapes under light stimulus. Chen et al. produced a graphene actuator with integrated-sensing function, whose shape changes will feedback real-timely (Chen et al., 2019). The reduced GO-paper (RGO-paper) in the actuator not only serves as an energy conversion material, but also functions as a sensing layer. The photothermal expansion strategy is a simple but efficient way for designing light-driven actuators.

### Photothermal Desorption

GO is sensitive to humidity due to the strong interaction between H_2_O and OCGs. When GO is combined with another material, which is inert to humidity, bending behavior could be observed during temperature changes. Water molecules escape from the GO upon photothermal effect, resulting in the distinct volume contraction of GO layer. However, the volume of the other material remains unchanged. The different volume changes of the two layer lead to a directional bending. Various humidity actuators have been made based on the reversible adsorption/desorption of water molecules (Han et al., 2015; Wang et al., 2019). Considering that the adsorption/desorption behaviors depend on the environment temperature, humidity-responsive material can be designed into photothermal desorption actuators. Han et al. coupled GO with RGO forming a competitive water absorption bilayer structure (Han D. D. et al., 2018). Upon light irradiation, GO/RGO converted light energy into heat, which triggers the desorption of water molecules from GO and leads to the deformation. Furthermore, actuators can be made based on the synergistic effect of photothermal expansion and desorption. The deformation becomes more obvious based on the dual-mode actuation. As a typical sample, Chen et al. proposed an actuator using GO and biaxially oriented poly-propylene (BOPP) (Chen L. et al., 2017). GO layer has a low CTE and a large coefficient of hydroscopic expansion (CHE), while BOPP has a high CTE and a relatively low CHE. Such complementary design of actuators shows better bending performance.

In addition to GO, many other materials can also interact with water molecules, illustrating adsorption/desorption properties. Thermally-active hydrogels can swell in water and shrink upon heating. However, the response time is relatively long, due to the poor heat conducting property. Considering graphene has a high thermal conductivity and good photothermal conversion efficiency, hydrogel usually combined with graphene forming photo-active hydrogel actuators, such as polydopamine (PDA) (Mu et al., 2015), elastin-like polypeptides (ELPs) (Wang et al., 2013), poly-ethylenimine (PEI) (Zhang and Tan, 2018), and poly N-isopropylacrylamide (PNIPAM) (Ma et al., 2018). Compared to pure hydrogel actuators, the response time of the actuators made by composite materials are quite fast.

### Photoisomerization

Azo dye molecules can change their shape upon alternate irradiation of UV light, according the *cis-tran* transform of molecular structure. Such photoisomerization kinetics are widely introduced in producing light-responsive actuator. Liquid crystals (LCs), of which mesogenic units can arrange in two separated phases, are inert to light. Macroscopic large-scale deformation can be achieved by the combination of these two materials. However, graphene has only one phase, so that the graphene layer remains unchanged during the photoisomerization process. On the other hand, high photothermal conversion efficiency of graphene also accelerates the photoisomerization progress. Cheng et al. produced a NIR-vis-UV light sensitive actuator by incorporation GO with LC and azobenzene dye (Cheng et al., 2015). In this work, GO can act as an excellent photothermal conversion reagent by absorbing visible and NIR light.

### Photo-Triggered Shape Memory Effect

Shape memory polymers (SMPs) can memorize a specific shape during fabrication. No matter if it is pressed, stretched or folded into any temporary structure, it will recover to the definite shape under certain stimuli, namely shape memory effect (SME). Photo-triggered SMP are also introduced to the actuation systems for their large-scale shape transformation and good mechanical property. Thermal-induced shape memory effect is the most common way to trigger the transformation of the polymer matrix. When combining such SMP with graphene, the actuation performance can be obtained through photothermal effect. Liang et al. proposed an actuator using graphene and thermoplastic polyurethane (TPU) materials, showing excellent light-triggered SME (Liang et al., 2009). Here, graphene functions as a light absorber and heat conductor. Besides, graphene also enhanced the mechanical property of TPU. Light-driven strategy has a high spatial resolution. The sequential recovery procedure is achieved by controlling the light spot position, avoiding self-interferences of complex shape actuation (Chen T.T. et al., 2017). In addition to making the SMP photo-active, the actuators based on graphene composite materials also indicate fast response property.

## Conclusion and Outlook

In conclusion, featuring unique mechanical, physical and chemical properties, graphene is favorable for designing light-responsive actuators. The underlying actuation mechanisms are summarized in this paper, including photothermal expansion, photothermal desorption, photoisomerization, and photo-induced SME. Actuators with fast response, large deflection, and complex shape change can be realized. However, most current studies are focusing on the material composition, fabrication process and fundamental actuation performance. More efforts should be made toward their practical usage in electronics, robotics and medical science, but it is still challenging at present. In addition, actuators can be coupled with various sensing, detecting, monitoring components for developing multifunctional and mature intelligent systems.

## Author Contributions

All authors listed have made a substantial, direct and intellectual contribution to the work, and approved it for publication.

### Conflict of Interest Statement

The authors declare that the research was conducted in the absence of any commercial or financial relationships that could be construed as a potential conflict of interest.
